# Perspectives on current models of Friedreich’s ataxia

**DOI:** 10.3389/fcell.2022.958398

**Published:** 2022-08-11

**Authors:** Simge Kelekçi, Abdullah Burak Yıldız, Kenan Sevinç, Deniz Uğurlu Çimen, Tamer Önder

**Affiliations:** School of Medicine, Koc University, Istanbul, Turkey

**Keywords:** iPSC (induced pluripotent stem cell), disease model cell, triplet repeat disease, frataxin, ataxia

## Abstract

Friedreich’s ataxia (FRDA, OMIM#229300) is the most common hereditary ataxia, resulting from the reduction of frataxin protein levels due to the expansion of GAA repeats in the first intron of the *FXN* gene. Why the triplet repeat expansion causes a decrease in Frataxin protein levels is not entirely known. Generation of effective FRDA disease models is crucial for answering questions regarding the pathophysiology of this disease. There have been considerable efforts to generate *in vitro* and *in vivo* models of FRDA. In this perspective article, we highlight studies conducted using FRDA animal models, patient-derived materials, and particularly induced pluripotent stem cell (iPSC)-derived models. We discuss the current challenges in using FRDA animal models and patient-derived cells. Additionally, we provide a brief overview of how iPSC-based models of FRDA were used to investigate the main pathways involved in disease progression and to screen for potential therapeutic agents for FRDA. The specific focus of this perspective article is to discuss the outlook and the remaining challenges in the context of FRDA iPSC-based models.

## Introduction

The prevalence of FRDA is 1 in 50,000 people, and the median age of onset is 10–15 years (Indelicato et al., 2020). Wheelchair use is generally necessary for around 15 years, and the average lifespan of FRDA patients is reported to be 36 years (Delatycki and Bidichandani, 2019). Approximately 96% of FRDA patients carry homozygous GAA triplet repeat expansion in the first intron of the frataxin (*FXN*) gene. The remaining 4% carry a heterozygous phenotype for GAA repeats with missense mutations in one allele and an expanded allele in the other (Al-Mahdawi et al., 2018). Normally, there are 5–33 GAA repeats in the first intron of the *FXN* gene; however, FRDA patients may have up to 1300 GAA repeats. Individuals with longer repeats show symptoms earlier and with increased severity (Delatycki et al., 2000; Delatycki and Bidichandani, 2019).

Friedreich’s ataxia is an autosomal recessive disorder with neurological and non-neurological manifestations. Neurological symptoms include progressive ataxia of gait and limbs, increased muscle tone, decrease in or loss of position sense and tendon reflexes, difficulty swallowing, and dysarthria (Filla et al., 1990; Parkinson et al., 2013). Additionally, neuroinflammation and upregulation of glial activation in the cerebellum and brainstem are observed (Apolloni et al., 2022; Khan et al., 2022). Non-neurological symptoms include hypertrophic cardiomyopathy, glucose intolerance, and diabetes mellitus (Campuzano et al., 1996; Gottesfeld, 2019). Foot deformity and scoliosis are the early signs of this disease in some cases. In others, cardiomyopathy is the first clinical symptom (Pandolfo, 2009). Diabetes mellitus usually develops in the later stage (De Michele et al., 1996).

GAA repeat expansions lead to a reduction in frataxin protein levels to 5–35% (Gellera et al., 2007; Abruzzo et al., 2013; Bürk, 2017; Vannocci et al., 2018). Why GAA repeat expansion leads to a reduction in frataxin protein levels and the role of frataxin in FRDA pathologies remain unclear. Likewise, the molecular pathology of cardiomyopathy in FRDA is not entirely elucidated, even though the most common cause of death is cardiomyopathy (Crombie et al., 2017). There are neither effective therapeutics for this disease nor any drugs that can slow its progression (Strawser et al., 2014). Therefore, modeling approaches are crucial. However, the selection of suitable models is an important step. The selected model should appropriately reflect FRDA pathophysiology.

Animal models have been nicely covered (Perdomini et al., 2013), whereas recent reviews about the molecular pathways involved in FRDA progression have been discussed in detail (Gottesfeld, 2019). In this perspective article, we focused on the challenges of FRDA *in vivo* (both non-complex and complex organisms) and patient-derived models and discussed iPSC-based models more comprehensively.

**FIGURE 1:**
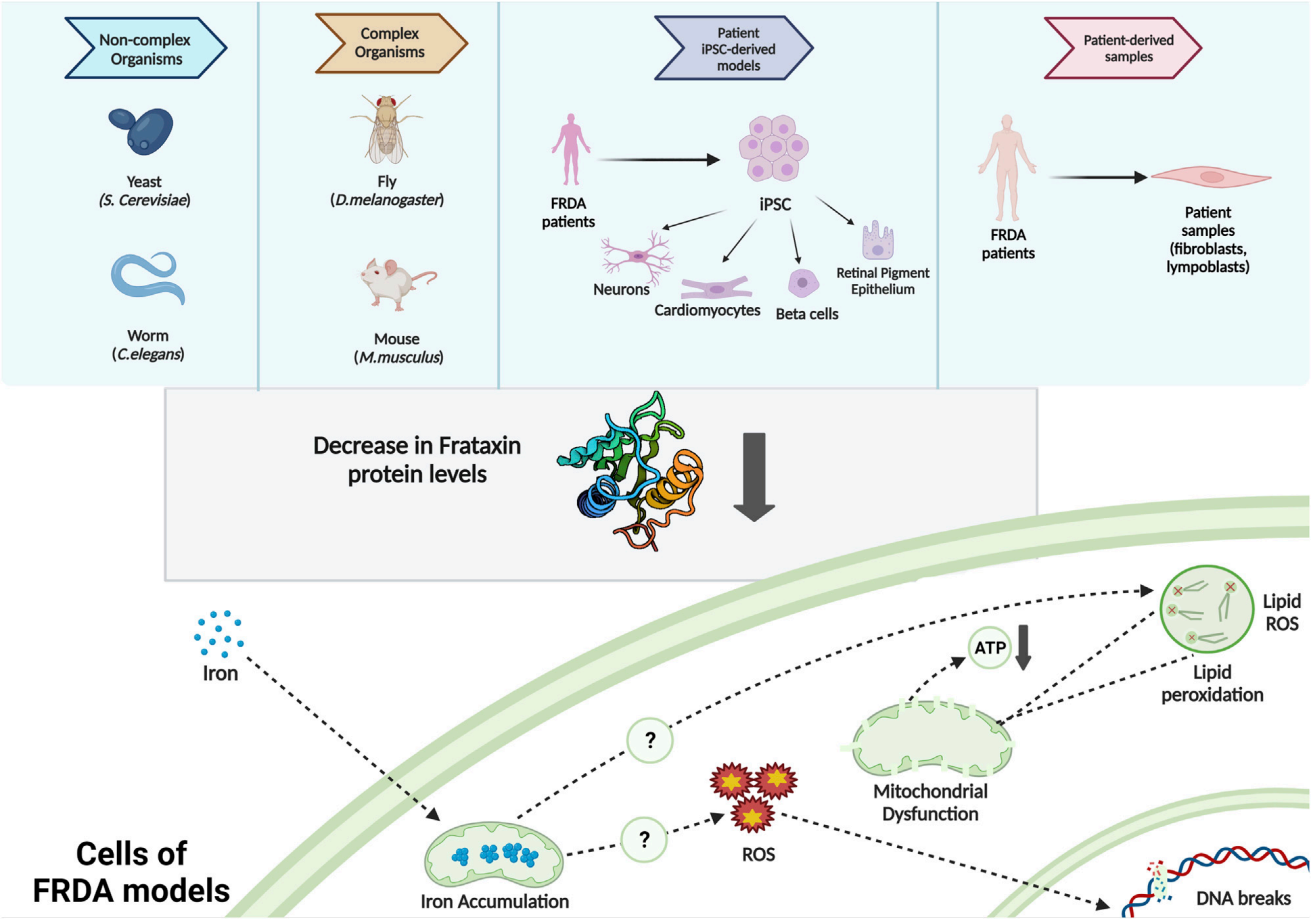
Commonly used FRDA models and the intracellular molecular phenotypes observed.

*In vivo* models of FRDA include yeast, nematode worm, fruit fly, and mice (Babcock et al., 1997; Radisky et al., 1999; Puccio et al., 2001; Al-Mahdawi et al., 2004; Vázquez-Manrique et al., 2006; Virmouni et al., 2014; Monnier et al., 2018), whereas FRDA *in vitro* models include patient-derived cells such as immortalized lymphoblastoid cells, primary fibroblasts (Li et al., 2016; Agro and Diaz-Nido, 2020; Misiorek et al., 2020; Johnson et al., 2021), FRDA-derived iPSCs (Angulo et al., 2021; Kelekci et al., 2021), and iPSC-based models such as neurons, cardiomyocytes, and beta cells (Crombie et al., 2016; Schreiber et al., 2019) (Figure 1). All these FRDA models need to imitate the symptoms of FRDA patients. For instance, they should be exhibiting a progression of sensory ataxia and/or cardiomyopathy due to reduced frataxin protein level, ideally as a result of GAA expansions in the first intron of the *FXN* gene (Perdomini et al., 2013).

## Challenges of Friedreich’s ataxia *in vivo*, and patient-derived models

The genetic basis of FRDA makes it challenging to study *in vivo*. In mice, knockout of the *FXN* gene is embryonically lethal (at E 6.5) (Cossee, 2000). Therefore, conditional knockout FRDA mouse models were generated. Expression of Cre recombinase was induced under *MCK* (muscle creatine kinase, a gene expressed in the heart cells) and *NSE* (neuron-specific enolase, a gene expressed in the neurons) promoters (Puccio et al., 2001; Puccio, 2009). Along with developing cardiomyopathy, MCK mice exhibited ISC enzyme deficiencies and iron accumulation at the early stages (Puccio, 2009; Simon et al., 2004). NSE mice developed progressive ataxia with a loss of proprioception (Simon et al., 2004; Perdomini et al., 2013). Additionally, a reversible frataxin knockdown mouse model was developed, and it had cardiac conduction defects, degeneration of dorsal root ganglia, and early mortality (Chandran et al., 2017). Although conditional models reproduce most of the characteristic features of FRDA, they cannot accurately model the effects of genetic background. Moreover, conditional models lead to a complete loss of frataxin, but in FRDA patients, partial frataxin deficiency is observed (Puccio, 2009; Perdomini et al., 2013).

GAA insertion models were also developed. Y4R and YG8R were generated by inserting expanded (GAA)_90_ and (GAA)_190_ alleles, respectively. Both express only human frataxin (Al-Mahdawi et al., 2004; Al-Mahdawi et al., 2006; Perdomini et al., 2013). The frataxin protein level was 57% in YG8R, which exhibited mild progressive motor coordination deficits (Al-Mahdawi et al., 2006; Clark et al., 2007; Martelli et al., 2012). Later, YG8sR mice were generated from YG8R. YG8sR mice have a single copy of the *FXN* transgene and a single (GAA)_120_ repeat expansion mutation. They exhibited behavioral deficits along with decreased expression of *FXN*, and reduced aconitase activity in brain regions (Anjomani Virmouni et al., 2015).

However, the short life span of *in vivo* models leads to incomplete development of pathological hallmarks. Also, although there is physiological accordance between humans and rodents, there are genomic differences that result in profound implications in disease modeling (Dawson et al., 2018). Therefore, further studies on FRDA animal models to overcome these challenges and thereby represent the disease phenotype better and faster are needed.

In 1999, human FRDA primary fibroblasts were used to understand whether this cell type is a reasonable model to study GAA expansions and to test potential therapeutic drugs. The fibroblasts had reduced levels of frataxin mRNA and were more sensitive to ROS-inducers (A. Wong, 1999). This study led FRDA fibroblasts and lymphoblastoid cells to be used in understanding FRDA phenotypes and investigating the effect of potential therapeutic agents. Although primary fibroblasts and lymphoblastoid cells are easily accessible, they are not the main cell types affected in the course of FRDA and they may not represent the phenotypes of FRDA adequately (Perdomini et al., 2013). Georges et al. (2019) showed that resveratrol, an antioxidant drug, and nicotinamide, vitamin B3, did not induce a significant increase in frataxin protein levels in FRDA iPSC-based neurons, although both drugs were shown to increase frataxin mRNA levels in fibroblasts and lymphoblastoid cells of FRDA patients. Additionally, one has to keep in mind that there are limited sources to work with fibroblasts because they do not grow indefinitely. Therefore, in this perspective article, we did not focus on studies where these patient-derived cells were used.

To summarize, model organisms and patient-derived cells can be used to model FRDA, but due to the technical and biological limitations, there is a need for more human FRDA-like models such as FRDA iPSC-based cells or organoids.

## Generation of patient-specific induced pluripotent stem cells

The discovery that exogenous expression of *OCT4, SOX2, KLF4,* and *c-MYC* can reprogram mammalian somatic cells back to an embryonic-like state (Takahashi and Yamanaka, 2006) enabled scientists to generate disease-specific pluripotent stem cell lines (Soldner and Jaenisch, 2018). As iPSCs can be autologous, they can replace embryonic stem cells in the field of clinical applications of cellular replacement therapies and screening for potential pharmaceuticals (Onder and Daley, 2012). The first iPSC-based disease model was familial amyotrophic lateral sclerosis (ALS) (Dimos et al., 2008). Since then, iPSC-based disease models have been developed to study a diverse set of neurological diseases, such as spinal muscular atrophy (SMA) (Ebert et al., 2009), Rett syndrome (Marchetto et al., 2010), and Friedreich’s ataxia (Ku et al., 2010).

### Impact of Friedreich’s ataxia-derived induced pluripotent stem cells and induced pluripotent stem cell-based models on Friedreich’s ataxia research

In 2010, iPSCs were generated from fibroblasts of FRDA patients for the first time. *FXN* downregulation observed in fibroblasts was found to be retained in the iPSCs (Ku et al., 2010). Since then, several additional FRDA patient-derived iPSCs were generated to study the pathophysiology of this disease (Liu et al., 2011; Bolotta et al., 2019; Schreiber et al., 2019; Dionisi et al., 2020; Mazzara et al., 2020; Angulo et al., 2021; Kelekci et al., 2021).

### Modeling GAA repeat instability with induced pluripotent stem cells

When the first FRDA-derived iPSCs were generated, genomic instability during reprogramming and culturing of iPSCs was observed (Ku et al., 2010; Sharma, 2002). The reason for GAA repeat instability in iPSCs was due to elevated expression levels of mismatch repair enzymes (MSH2, MSH3, and MSH6) (Jintang Du et al., 2012). Defects in replication fork progression and stalling in the 3′–5′ direction were also observed during the replication of *FXN* (Gerhardt et al., 2016). Interestingly, treatment of FRDA fibroblasts with certain epigenetic drugs such as sodium butyrate (NaB), an HDAC class I inhibitor, and Parnate, a LSD1 inhibitor, led to decreased repeat instability during reprogramming. These compounds increased *FXN* gene expressions (Polak et al., 2016).

Recently, DNA methylation patterns on GAA repeats were studied. FRDA-specific differentially methylated regions (DMRs) were found to be closer to GAA repeats in FRDA iPSC-based models. Interestingly, the prevalence of *FXN* genes that lack FRDA-specific hypomethylation (unmethylated epialleles) was found to be a predictor of *FXN* expression and age of onset of FRDA (Rodden et al., 2021). However, the reason for the absence of unmethylated epialleles in GAA repeats near the *FXN* gene and the effect of methylation on GAA expansion remains to be elucidated.

Considering all these, further studies are necessary to understand the mechanisms behind the repeat expansions and repeat instability observed in FRDA iPSCs. Specifically, the effect of mismatch repair enzymes, the role of methylation in the repeats, and the reason for replication fork stalling should be further investigated in the future.

### Modeling of Friedreich’s ataxia phenotypes using induced pluripotent stem cell-based models

Decrease in frataxin expression causes iron accumulation, impaired iron–sulfur cluster biogenesis, increased oxidative stress, mitochondrial dysfunctioning (Calap-Quintana et al., 2018; Gonzalez-Cabo & Palau, 2013; Santos et al., 2010), and ferroptosis (Cotticelli et al., 2019; La Rosa et al., 2020; Turchi et al., 2020; Terzi et al., 2021). These phenotypes were also recapitulated in iPSC-based *in vitro* models of FRDA (Schreiber et al., 2019). Here, we highlight recent studies utilizing FRDA iPSC-based models (Table 1).

**TABLE 1:** FRDA iPSC-based models and main findings observed.

FRDA iPSC-based models	Main phenotypes observed	References
FRDA iPSC-derived neurons	Delayed development of full electrophysiological functionality, reduced mitochondrial potential	Hick et al. (2013), Codazzi et al. (2016)
	Enhanced cleavage of initiator caspase-9 and effector caspase-3 activation	Igoillo-Esteve et al. (2015)
	Quantitative proteomic analysis of HDAC inhibitor treatment	Shan et al. (2014)
	Promising effects of Syn-TEFs	Erwin et al. (2017)
	Transcriptional profiling of isogenic and FRDA iPSC-derived neurons and investigation of the effect of HDAC inhibitors	Lai et al. (2019)
	Generation of a novel FRDA iPSC-derived neuronal reporter system and screening of compounds using this system	Schreiber et al. (2022)
FRDA iPSC-derived cardiomyocytes	Characteristics of respiration-compromised mitochondria, mitochondrial iron accumulation	Hick et al. (2013)
	Disorganized mitochondrial network, mitochondrial DNA depletion, hypertrophic cardiac stress responses, use of FRDA iPSC-derived cardiomyocytes as a drug screening platform	Lee et al. (2014), Lee et al. (2016)
	Calcium signaling impairment	Crombie et al. (2017)
	Promising effects of Syn-TEFs	Erwin et al. (2017)
	Observation of lipid droplets	J. Li et al. (2019)
	Excision of repeats via zinc finger nucleases and upregulation of *FXN*	J. Li et al. (2019)
	Strong positive correlation between the contractility/developed force and *FXN* expression is observed in FRDA iPSC-derived cardiomyocytes	A. O. T. Wong et al. (2019)
	Hepcidin (HAMP)–ferroportin (FPN) axis impaired in FRDA iPSC-derived cardiomyocytes	Bolotta et al. (2019)
FRDA iPSC-derived endothelial cells	Investigation of senescence and the relation between *FXN* expression and pulmonary hypertension	Culley et al. (2021)
FRDA iPSC-derived beta cells	Low *FXN* levels in FRDA iPSC-derived beta cells, upregulation of it *via* glucagon-1-peptide treatment	Igoillo-Esteve et al. (2015), Igoillo-Esteve et al. (2020)

FRDA iPSC-based neurons exhibited reduced mitochondrial transcripts and altered expression levels in transcripts involved in ECM organization and focal adhesion (Lai et al., 2019). Primary proprioceptive neurons differentiated from FRDA iPSCs had reduced expression of proprioceptor-specific markers and exhibited shorter survival *in vitro* (Dionisi et al., 2020).

FRDA iPSC-derived cardiomyocytes (FRDA iPSC-Cms) showed decreased levels of frataxin and mitochondrial potential and impaired functioning of mitochondria (Hick et al., 2013). Lee et al. (2014) exposed FRDA iPSC-Cms to iron-overloading conditions, and this resulted in hypertrophic cardiac stress responses, iron accumulation, and an increase in ROS formation. FRDA iPSC-Cms had calcium handling deficiencies (Crombie et al., 2017). They also exhibited lipid droplet accumulation (J. Li et al., 2019).

FRDA iPSCs were differentiated into endothelial cells, as well. FRDA iPSC-based endothelial cells (iPSC-ECs) exhibited decreased proliferative activity along with an increase in the expression of cellular senescence markers. Forced *FXN* expression in FRDA iPSC-ECs did not rescue this phenotype, showing that this senescence phenotype is irreversible (Culley et al., 2021).

FRDA iPSC-based β cells were generated in 2019, and those cells had lower *FXN* gene expression. However, FRDA iPSC-based β cells had functional mitochondria and functional secretion of glucose-induced insulin (Igoillo-Esteve et al., 2020).

### Therapeutic solutions for Friedreich’s ataxia with the help of induced pluripotent stem cell-based technologies

Heterochromatin formation around the *FXN* gene is the major reason for the downregulation of the *FXN* gene in FRDA (Chutake et al., 2014; Kumari et al., 2011). Histone deacetylation, DNA methylation, and heterochromatin mark (H3K9me3 and H3K27me3) were detected around the *FXN* gene (Al-Mahdawi et al., 2008). Therefore, epigenetic regulators such as HDAC inhibitors have been studied using FRDA iPSC-based *in vitro* models (Zhang et al., 2019). HDAC inhibitor treatment of FRDA iPSC-based neurons resulted in a significant increase in *FXN* levels along with a decrease in oxidative stress response (Codazzi et al., 2016). More recently, large-scale chemical screens on FRDA iPSC-based neuronal progenitor cells containing a *FXN* reporter system revealed HDAC inhibitors as the only compounds that could upregulate *FXN* levels (Schreiber et al., 2022).

Recent advances in genome editing enabled the correction of expanded alleles in FRDA. Three-fold higher expression of frataxin in both mRNA and protein levels was observed in FRDA iPSC-based neurons after zinc finger nuclease (ZFN)-mediated excision of expanded GAA repeat regions (Li et al., 2015). In addition, an increase in aconitase activity and ATP levels was observed (Li et al., 2015). Similarly, GAA expanded repeats were excised using ZFNs in FRDA iPSC-based cardiomyocytes, which resulted in the upregulation of frataxin expression, a decrease in lipid droplet accumulation, and a decrease in the expression of cardiac hypertrophy-related genes (J. Li et al., 2019). Recently, dorsal root ganglia organoid-derived sensory neurons were generated from FRDA iPSCs. When the entire *FXN* intron 1 was excised using CRISPR-Cas9, cellular and molecular deficits observed in these organoids were rescued. This effect was not observed when only the expanded regions were removed in those organoids (Mazzara et al., 2020). All of these studies show that nuclease-mediated removal systems can be used to rescue FRDA phenotypes in the future.

Recently, repeat-targeted nucleic acids (L. Li et al., 2016; Shen et al., 2019; Shen et al., 2020) and synthetic transcription elongation factors (Syn-TEFs) (Erwin et al., 2017) were used to elevate *FXN* expression in FRDA iPSC-based models. In addition, particle-mediated delivery of frataxin-encoding plasmid DNA has been used to increase *FXN* levels in FRDA iPSC-based sensory neurons (Czuba-Wojnilowicz et al., 2020). Oligonucleotides targeting 5′ and/or 3’ untranslated regions of the *FXN* transcript increased *FXN* mRNA and protein levels in FRDA iPSC-based neuronal progenitor cells (Belbellaa et al., 2020). Taken together, these studies show that overexpression of *FXN* can have therapeutic potential, but further optimization is required.

## Discussion and future perspectives on Friedreich’s ataxia models

FRDA is a rare neurodegenerative disorder, mediated by triplet repeat expansion which results in the downregulation of frataxin. In the recent years, there have been considerable efforts to find a promising drug for FRDA. Therapeutic approaches to augment frataxin production or modulate mitochondrial malfunctioning, increase ROS production, and Nrf2 activation have been used in phase II clinical trials (Rodden & Lynch, 2021). Additionally, there are many studies addressing potentially promising therapeutic approaches for FRDA pathophysiology. For instance, smoothened agonist (SAG) treatment in *FXN* knockdown human astrocytes resulted in increased neuron viability, neurite length, and synapse formation when these cells were injected into the mice brain (Vicente-Acosta et al., 2022). Additionally, Cur@SF, which are nanospheres containing curcumin in silk fibroins, reduced the levels of ROS in FRDA patient-derived fibroblasts and reduced FRDA phenotypes in YG8R mice models (Xu et al., 2022). Lastly, after eight single FDA-approved drugs were tested on FRDA fibroblasts, dimethyl fumarate and resveratrol treatments were shown to increase *FXN* mRNA levels. The combination of these two drugs also increased rotarod performance in FRDA mice models (Abeti et al., 2022). However, all these promising drugs should be further tested in FRDA iPSC-derived neurons and cardiomyocytes as these cells are the main cell types affected in FRDA.

In this perspective article, we have discussed recent models of FRDA and their contributions to identify molecular mechanisms in FRDA development and develop potential therapeutics through drug screening and gene therapy studies. Animal models are composed of nematode worms, fruit flies, and mice. Many of these models were successful in recapitulating the FRDA phenotypes *in vivo*. However, the general concern about the animal models of FRDA is the difficulty in creating such models that are able to exhibit all FRDA-related symptoms and the cellular phenotypes due to reduced *FXN* levels resulting from GAA repeat expansion. *In vitro* models include primary cells, genetically modified cell lines (Perdomini et al., 2013), iPSC-based cells (Schreiber et al., 2019), and, recently, organoids (Mazzara et al., 2020). Consideration of models using primary patient-derived fibroblasts and lymphoblastoid cells remains to be controversial because these cells are disease-irrelevant. Differentiated FRDA-derived iPSC-based models exhibit gene expression profiles that are significantly different from the isogenic iPSC-based models created and show similar phenotypes observed in patients’ cells (Lai et al., 2019). Therefore, FRDA iPSC-based models can be considered promising models for future FRDA studies. However, FRDA-derived iPSC *in vitro* models have certain limitations as well. First is the repeat instability observed during the reprogramming and culturing duration of FRDA-derived iPSCs. This limitation may potentially be overcome by using therapeutic agents that may prevent this instability. Another challenge is the lack of paired isogenic lines. In the case of FRDA-derived iPSCs, isogenic derivatives can be created simply *via* the excision of repeats. Lastly, it may not be possible to study tissue and organ level phenotypes observed in FRDA patients *via* 2D differentiated cells. iPSC-based organoids provide an alternative in this regard. Nevertheless, thanks to FRDA iPSC-based models, the reasons behind GAA repeat expansion and mechanisms of disease-relevant phenotypes were studied thoroughly and efficient therapies using CRISPR/Cas9 to increase *FXN* expression were investigated over the past decades. However, there are still many open questions that need to be addressed and challenges to overcome.

## Data Availability

The original contributions presented in the study are included in the article/supplementary material; further inquiries can be directed to the corresponding authors.

## References

[B1] Abeti R. Jasoliya M. Al-Mahdawi S. Pook M. Gonzalez-Robles C. Hui C. K. (2022). A drug combination rescues frataxin-dependent neural and cardiac pathophysiology in FA models. Front. Mol. Biosci. 9, 830650. 10.3389/fmolb.2022.830650 35664670 PMC9160322

[B2] Abruzzo P. M. Marini M. Bolotta A. Malisardi G. Manfredini S. Ghezzo A. (2013). Frataxin mRNA isoforms in FRDA patients and normal subjects: effect of tocotrienol supplementation. Biomed. Res. Int., 276808. 10.1155/2013/276808 24175286 PMC3794619

[B3] Agro M. Diaz-Nido J. (2020). Effect of mitochondrial and cytosolic *FXN* isoform expression on mitochondrial dynamics and metabolism. Int. J. Mol. Sci. 21 (21), E8251. 10.3390/ijms21218251 33158039 PMC7662637

[B4] Al-Mahdawi S. Ging H. Bayot A. Cavalcanti F. La Cognata V. Cavallaro S. (2018). Large interruptions of GAA repeat expansion mutations in friedreich ataxia are very rare. Front. Cell. Neurosci. 12, 443. 10.3389/fncel.2018.00443 30519163 PMC6258883

[B5] Al-Mahdawi S. Pinto R. M. Ruddle P. Carroll C. Webster Z. Pook M. (2004). GAA repeat instability in friedreich ataxia YAC transgenic mice. Genomics 84, 301–310. 10.1016/j.ygeno.2004.04.003 15233994

[B6] Al-Mahdawi S. Pinto R. M. Varshney D. Lawrence L. Lowrie M. B. Hughes S. (2006). GAA repeat expansion mutation mouse models of friedreich ataxia exhibit oxidative stress leading to progressive neuronal and cardiac pathology. Genomics 88 (5), 580–590. 10.1016/j.ygeno.2006.06.015 16919418 PMC2842930

[B87] Al-Mahdawi S. Pinto R. M. Ismail O. Varshney D. Lymperi S. Sandi C. (2008). The Friedreich ataxia GAA repeat expansion mutation induces comparable epigenetic changes in human and transgenic mouse brain and heart tissues. Hum. Mol. Genet. 17 (5), 735–746. 10.1093/hmg/ddm346 18045775

[B7] Angulo M. B. Yang J. Argenziano M. A. Bertalovitz A. C. Beidokhti M. N. McDonald T. V. (2021). Generation of a Friedreich's ataxia patient-derived iPSC line USFi001-A. Stem Cell Res. 54, 102399. 10.1016/j.scr.2021.102399 34034220

[B8] Anjomani Virmouni S. Ezzatizadeh V. Sandi C. Sandi M. Al-Mahdawi S. Chutake Y. (2015). A novel GAA-repeat-expansion-based mouse model of Friedreich's ataxia. Dis. Model. Mech. 8 (3), 225–235. 10.1242/dmm.018952 25681319 PMC4348561

[B9] Apolloni S. Milani M. D'Ambrosi N. (2022). Neuroinflammation in friedreich's ataxia. Int. J. Mol. Sci. 23 (11), 6297. 10.3390/ijms23116297 35682973 PMC9181348

[B10] Babcock M. De Silva D. Oaks R. Davis-Kaplan S. Jiralerspong S. Montermini L. (1997). Regulation of mitochondrial iron accumulation by Yfh1p, a putative homolog of Frataxin. Science 276, 1709–1712. 10.1126/science.276.5319.1709 9180083

[B11] Belbellaa B. Reutenauer L. Messaddeq N. Monassier L. Puccio H. (2020). High levels of frataxin overexpression lead to mitochondrial and cardiac toxicity in mouse models. Mol. Ther. Methods Clin. Dev. 19, 120–138. 10.1016/j.omtm.2020.08.018 33209958 PMC7648087

[B12] Bolotta A. Abruzzo P. M. Baldassarro V. A. Ghezzo A. Scotlandi K. Marini M. (2019). New insights into the hepcidin-ferroportin Axis and iron homeostasis in iPSC-derived cardiomyocytes from friedreich’s ataxia patient. Oxid. Med. Cell. Longev., 7623023. 10.1155/2019/7623023 31049138 PMC6458886

[B13] Bürk K. (2017). Friedreich Ataxia: current status and future prospects. Cerebellum Ataxias 4, 4. 10.1186/s40673-017-0062-x PMC5383992 28405347

[B14] Campuzano V. Montermini L. Moltò M. D. Pianese L. Cossée M. Cavalcanti F. (1996). Friedreich’s ataxia: autosomal recessive disease caused by an intronic GAA triplet repeat expansion. Science 271, 1423–1427. 10.1126/science.271.5254.1423 8596916

[B15] Chandran V. Gao K. Swarup V. Versano R. Dong H. Jordan M. C. (2017). Inducible and reversible phenotypes in a novel mouse model of friedreich's ataxia. Elife 6, e30054. 10.7554/eLife.30054 29257745 PMC5736353

[B88] Chutake Y. K. Lam C. Costello W. N. Anderson M. Bidichandani S. I. (2014). Epigenetic promoter silencing in *Friedreich ataxia* is dependent on repeat length. Ann. Neurol. 76 (4), 522–528. 10.1002/ana.24249 25112975 PMC4191993

[B16] Clark R. M. De Biase I. Malykhina A. P. Al-Mahdawi S. Pook M. Bidichandani S. I. (2007). The GAA triplet-repeat is unstable in the context of the human FXN locus and displays age-dependent expansions in cerebellum and DRG in a transgenic mouse model. Hum. Genet. 120 (5), 633–640. 10.1007/s00439-006-0249-3 17024371

[B17] Codazzi F. Hu A. Rai M. Donatello S. Salerno Scarzella F. Mangiameli E. (2016). Friedreich ataxia-induced pluripotent stem cell-derived neurons show a cellular phenotype that is corrected by a benzamide HDAC inhibitor. Hum. Mol. Genet. 25 (22), 4847–4855. 10.1093/hmg/ddw308 28175303

[B18] Cossee M. Puccio H. GAnsmuller A. Koutnikova H. Dierich A. LeMeurM. (2000). Inactivation of the Friedreich ataxia mouse gene leads to early embryonic lethality without iron accumulation. Hum. Mol. Genet. 9, 1219–1226. 10.1093/hmg/9.8.1219 10767347

[B19] Cotticelli M. G. Xia S. Lin D. Lee T. Terrab L. Wipf P. (2019). Ferroptosis as a novel therapeutic target for friedreich's ataxia. J. Pharmacol. Exp. Ther. 369 (1), 47–54. 10.1124/jpet.118.252759 30635474

[B20] Crombie D. E. Curl C. L. Raaijmakers A. J. A. Sivakumaran P. Kulkarni T. Wong R. C. B. (2017). Friedreich’s ataxia induced pluripotent stem cell-derived cardiomyocytes display electrophysiological abnormalities and calcium handling deficiency. Aging 9, 1440–1452. 10.18632/aging.101247 28562313 PMC5472743

[B21] Crombie D. E. Curl C. L. Raaijmakers A. J. Sivakumaran P. Kulkarni T. Wong R. C. (2017). Friedreich's ataxia induced pluripotent stem cell-derived cardiomyocytes display electrophysiological abnormalities and calcium handling deficiency. Aging (Albany NY) 9 (5), 1440–1452. 10.18632/aging.101247 28562313 PMC5472743

[B22] Crombie D. E. Pera M. F. Delatycki M. B. Pebay A. (2016). Using human pluripotent stem cells to study friedreich ataxia cardiomyopathy. Int. J. Cardiol. 212, 37–43. 10.1016/j.ijcard.2016.03.040 27019046

[B23] Culley M. K. Zhao J. Tai Y. Y. Tang Y. Perk D. Negi V. (2021). Frataxin deficiency promotes endothelial senescence in pulmonary hypertension. J. Clin. Invest. 131 (11), 136459. 10.1172/JCI136459 33905372 PMC8159699

[B89] Czuba-Wojnilowicz E. Viventi S. Howden S. E. Maksour S. Hulme A. E. Cortez-Jugo C. (2020). Particle-mediated delivery of frataxin plasmid to a human sensory neuronal model of Friedreich's ataxia. Biomater. Sci. 8 (9), 2398–2403. 10.1039/c9bm01757g 32270790

[B24] Dawson T. M. Golde T. E. Lagier-Tourenne C. (2018). Animal models of neurodegenerative diseases. Nat. Neurosci. 21, 1370–1379. 10.1038/s41593-018-0236-8 30250265 PMC6615039

[B25] De Michele G. DiMaio L. Filla A. Majello M. Cocozza S. Cavalcanti F. (1996). Childhood onset of friedreich ataxia: a clinical and genetic study of 36 cases. Neuropediatrics 27 (1), 3–7. 10.1055/s-2007-973740 8677022

[B26] Delatycki M. B. Bidichandani S. I. (2019). “Friedreich ataxia- pathogenesis and implications for therapies,” in Neurobiology of disease. 10.1016/j.nbd.2019.104606 31494282

[B27] Delatycki M. B. Williamson R. Forrest S. M. (2000). Friedreich ataxia: an overview. J. Med. Genet. 37 (1), 1–8. 10.1136/jmg.37.1.1 10633128 PMC1734457

[B28] Dimos J. T. Rodolfa K. T. Niakan K. K. Weisenthal L. M. Mitsumoto H. Chung W. (2008). Induced pluripotent stem cells generated from patients with ALS can be differentiated into motor neurons. Science 321 (5893), 1218–1221. 10.1126/science.1158799 18669821

[B29] Dionisi C. Rai M. Chazalon M. Schiffmann S. N. Pandolfo M. (2020). Primary proprioceptive neurons from human induced pluripotent stem cells: a cell model for afferent ataxias. Sci. Rep. 10 (1), 7752. 10.1038/s41598-020-64831-6 32385372 PMC7210273

[B30] Du J. Campau E. Soragni E. Ku S. Puckett J. W. Dervan P. B. (2012). Role of mismatch repair enzymes in GAA·TTC triplet-repeat expansion in friedreich ataxia induced pluripotent stem cells. J. Biol. Chem. 287, 29861–29872. 10.1074/jbc.M112.391961 22798143 PMC3436184

[B31] Ebert A. D. Yu J. Rose F. F. Jr. Mattis V. B. Lorson C. L. Thomson J. A. (2009). Induced pluripotent stem cells from a spinal muscular atrophy patient. Nature 457 (7227), 277–280. 10.1038/nature07677 19098894 PMC2659408

[B32] Erwin G. S. Grieshop M. P. Ali A. Qi J. Lawlor M. Kumar D. (2017). Synthetic transcription elongation factors license transcription across repressive chromatin. Science 358 (6370), 1617–1622. 10.1126/science.aan6414 29192133 PMC6037176

[B33] Filla A. DeMichele G. Caruso G. Marconi R. Campanella G. (1990). Genetic data and natural history of friedreich's disease: a study of 80 Italian patients. J. Neurol. 237 (6), 345–351. 10.1007/bf00315657 2277267

[B34] Gellera C. Castellotti B. Mariotti C. Mineri R. Seveso V. DiDonato S. (2007). Frataxin gene point mutations in italian friedreich ataxia patients. Neurogenetics 8, 289–299. 10.1007/s10048-007-0101-5 17703324

[B35] Georges P. Boza-Moran M. G. Gide J. Pêche G. A. Forêt B. Bayot A. (2019). Induced pluripotent stem cells-derived neurons from patients with friedreich ataxia exhibit differential sensitivity to resveratrol and nicotinamide. Sci. Rep. 9, 14568. 10.1038/s41598-019-49870-y 31601825 PMC6787055

[B36] Gerhardt J. Bhalla A. D. Butler J. S. Puckett J. W. Dervan P. B. Rosenwaks Z. (2016). Stalled DNA replication forks at the endogenous GAA repeats drive repeat expansion in friedreich’s ataxia cells. Cell Rep. 16, 1218–1227. 10.1016/j.celrep.2016.06.075 27425605 PMC5028224

[B37] Gonzalez-Cabo P. Palau F. (2013). Mitochondrial pathophysiology in friedreich's ataxia. J. Neurochem. 126, 53–64. 10.1111/jnc.12303 23859341

[B38] Gottesfeld J. M. (2019). Molecular mechanisms and therapeutics for the GAA·TTC expansion disease friedreich ataxia. Neurotherapeutics 16, 1032–1049. 10.1007/s13311-019-00764-x 31317428 PMC6985418

[B39] Hick A. Wattenhofer-Donze M. Chintawar S. Tropel P. Simard J. P. Vaucamps N. (2013). Neurons and cardiomyocytes derived from induced pluripotent stem cells as a model for mitochondrial defects in friedreich's ataxia. Dis. Model. Mech. 6 (3), 608–621. 10.1242/dmm.010900 23136396 PMC3634645

[B40] Igoillo-Esteve M. Gurgul-Convey E. Hu A. Romagueira Bichara Dos Santos L. Abdulkarim B. Chintawar S. (2015). Unveiling a common mechanism of apoptosis in beta-cells and neurons in friedreich's ataxia. Hum. Mol. Genet. 24 (8), 2274–2286. 10.1093/hmg/ddu745 25552656

[B41] Igoillo-Esteve M. Oliveira A. F. Cosentino C. Fantuzzi F. Demarez C. Toivonen S. (2020). Exenatide induces frataxin expression and improves mitochondrial function in friedreich ataxia. JCI Insight 5 (2), 134221. 10.1172/jci.insight.134221 31877117 PMC7098728

[B42] Indelicato E. Nachbauer W. Eigentler A. Amprosi M. Matteucci Gothe R. Giunti P. (2020). Onset features and time to diagnosis in friedreich's Ataxia. Orphanet J. Rare Dis. 15 (1), 198. 10.1186/s13023-020-01475-9 32746884 PMC7397644

[B43] Johnson J. Mercado-Ayon E. Clark E. Lynch D. Lin H. (2021). Drp1-dependent peptide reverse mitochondrial fragmentation, a homeostatic response in friedreich ataxia. Pharmacol. Res. Perspect. 9 (3), e00755. 10.1002/prp2.755 33951329 PMC8099044

[B44] Kelekci S. Ugurlu-Cimen D. Demir A. B. Ozcimen B. Burak Yildiz A. Batuhan Karakus M. (2021). Generation of transgene-free iPSC lines from three patients with friedreich's ataxia (FRDA) carrying GAA triplet expansions in the first intron of *FXN* gene. Stem Cell Res. 54, 102438. 10.1016/j.scr.2021.102438 34214898

[B45] Khan W. Corben L. A. Bilal H. Vivash L. Delatycki M. B. Egan G. F. (2022). Neuroinflammation in the cerebellum and brainstem in friedreich ataxia: An [18F]-FEMPA PET study. Mov. Disord. 37 (1), 218–224. 10.1002/mds.28825 34643298

[B46] Ku S. Soragni E. Campau E. Thomas E. A. Altun G. Laurent L. C. (2010). Friedreich’s ataxia induced pluripotent stem cells model intergenerational GAATTC triplet repeat instability. Cell Stem Cell 7, 631–637. 10.1016/j.stem.2010.09.014 21040903 PMC2987635

[B90] Kumari D. Biacsi R. E. Usdin K. (2011). Repeat expansion affects both transcription initiation and elongation in friedreich ataxia cells. J. Biol. Chem. 286 (6), 4209–4215. 10.1074/jbc.M110.194035 21127046 PMC3039332

[B47] La Rosa P. Petrillo S. Fiorenza M. T. Bertini E. S. Piemonte F. (2020). Ferroptosis in friedreich's ataxia: a metal-induced neurodegenerative disease. Biomolecules 10 (11), E1551. 10.3390/biom10111551 33202971 PMC7696618

[B48] Lai J. I. Nachun D. Petrosyan L. Throesch B. Campau E. Gao F. (2019). Transcriptional profiling of isogenic friedreich ataxia neurons and effect of an HDAC inhibitor on disease signatures. J. Biol. Chem. 294, 1846–1859. 10.1074/jbc.RA118.006515 30552117 PMC6369281

[B49] Lee Y. K. Ho P. W. Schick R. Lau Y. M. Lai W. H. Zhou T. (2014). Modeling of Friedreich ataxia-related iron overloading cardiomyopathy using patient-specific-induced pluripotent stem cells. Pflugers Arch. 466 (9), 1831–1844. 10.1007/s00424-013-1414-x 24327207

[B50] Li J. Rozwadowska N. Clark A. Fil D. Napierala J. S. Napierala M. (2019). Excision of the expanded GAA repeats corrects cardiomyopathy phenotypes of iPSC-derived friedreich's ataxia cardiomyocytes. Stem Cell Res. 40, 101529. 10.1016/j.scr.2019.101529 31446150 PMC6853280

[B51] Li Y. Polak U. Clark A. D. Bhalla A. D. Chen Y. Y. Li J. (2016). Establishment and maintenance of primary fibroblast repositories for rare diseases-friedreich's ataxia example. Biopreserv. Biobank. 14 (4), 324–329. 10.1089/bio.2015.0117 27002638 PMC4991587

[B52] Liu J. Verma P. J. Evans-Galea M. V. Delatycki M. B. Michalska A. Leung J. (2011). Generation of induced pluripotent stem cell lines from friedreich ataxia patients. Stem Cell Rev. Rep. 7, 703–713. 10.1007/s12015-010-9210-x 21181307

[B91] Li Y. Polak U. Bhalla A. D. Rozwadowska N. Butler J. S. Lynch D. R. (2015). Excision of Expanded GAA Repeats Alleviates the Molecular Phenotype of Friedreich’s Ataxia. Mol. Ther. 23 (6), 1055–1065. 10.1038/mt.2015.41 25758173 PMC4817761

[B53] Marchetto M. C. Carromeu C. Acab A. Yu D. Yeo G. W. Mu Y. (2010). A model for neural development and treatment of Rett syndrome using human induced pluripotent stem cells. Cell 143 (4), 527–539. 10.1016/j.cell.2010.10.016 21074045 PMC3003590

[B54] Martelli A. Napierala M. Puccio H. (2012). Understanding the genetic and molecular pathogenesis of friedreich's ataxia through animal and cellular models. Dis. Model. Mech. 5 (2), 165–176. 10.1242/dmm.008706 22382366 PMC3291638

[B55] Mazzara P. G. Muggeo S. Luoni M. Massimino L. Zaghi M. Valverde P. T. (2020). Frataxin gene editing rescues friedreich's ataxia pathology in dorsal root ganglia organoid-derived sensory neurons. Nat. Commun. 11 (1), 4178. 10.1038/s41467-020-17954-3 32826895 PMC7442818

[B56] Misiorek J. O. Schreiber A. M. Urbanek-Trzeciak M. O. Jazurek-Ciesiolka M. Hauser L. A. Lynch D. R. (2020). A comprehensive transcriptome analysis identifies *FXN* and BDNF as novel targets of miRNAs in friedreich's ataxia patients. Mol. Neurobiol. 57 (6), 2639–2653. 10.1007/s12035-020-01899-1 32291635 PMC7253519

[B57] Monnier V. Llorens J. V. Navarro J. A. (2018). Impact of drosophila models in the study and treatment of friedreich’s ataxia. Int. J. Mol. Sci. 19, E1989. 10.3390/ijms19071989 29986523 PMC6073496

[B58] Onder T. T. Daley G. Q. (2012). New lessons learned from disease modeling with induced pluripotent stem cells. Curr. Opin. Genet. Dev. 22 (5), 500-508. 10.1016/j.gde.2012.05.005 22749051 PMC3489983

[B59] Pandolfo M. (2009). Friedreich ataxia: the clinical picture. J. Neurol. 256, 3–8. 10.1007/s00415-009-1002-3 19283344

[B60] Parkinson M. H. Boesch S. Nachbauer W. Mariotti C. Giunti P. (2013). Clinical features of friedreich's ataxia: classical and atypical phenotypes. J. Neurochem. 126, 103–117. 10.1111/jnc.12317 23859346

[B61] Perdomini M. Hick A. Puccio H. Pook M. A. (2013). Animal and cellular models of friedreich ataxia. J. Neurochem. 126, 65–79. 10.1111/jnc.12219 23859342

[B62] Polak U. Li Y. Butler J. S. Napierala M. (2016). Alleviating GAA repeat induced transcriptional silencing of the friedreich’s ataxia gene during somatic cell reprogramming. Stem Cells Dev. 25, 1788–1800. 10.1089/scd.2016.0147 27615158 PMC5155629

[B63] Puccio H. (2009). Multicellular models of friedreich ataxia. J. Neurol. 256 Suppl 1, 18–24. 10.1007/s00415-009-1004-1 19283346

[B64] Puccio H. Simon D. Cossée M. Criqui-Filipe P. Tiziano F. Melki J. (2001). Mouse models for Friedreich ataxia exhibit cardiomyopathy, sensory nerve defect and Fe-S enzyme deficiency followed by intramitochondrial iron deposits. Nat. Genet. 27, 181–186. 10.1038/84818 11175786

[B65] Radisky D. C. Babcock M. C. Kaplan J. (1999). The yeast frataxin homologue mediates mitochondrial iron efflux: evidence for a mitochondrial iron cycle. J. Biol. Chem. 274, 4497–4499. 10.1074/jbc.274.8.4497 9988680

[B66] Rodden L. N. Chutake Y. K. Gilliam K. Lam C. Soragni E. Hauser L. (2021). Methylated and unmethylated epialleles support variegated epigenetic silencing in Friedreich ataxia. Hum. Mol. Genet. 29 (23), 3818–3829. 10.1093/hmg/ddaa267 33432325 PMC7861014

[B67] Rodden L. N. Lynch D. R. (2021). Designing phase II clinical trials in friedreich ataxia. Expert Opin. Emerg. Drugs 26 (4), 415–423. 10.1080/14728214.2021.1998452 34693848

[B69] Santos R. Lefevre S. Sliwa D. Seguin A. Camadro J. M. Lesuisse E. (2010). Friedreich ataxia: molecular mechanisms, redox considerations, and therapeutic opportunities. Antioxid. Redox Signal. 13 (5), 651–690. 10.1089/ars.2009.3015 20156111 PMC2924788

[B70] Schreiber A. M. Li Y. Chen Y. Napierala J. S. Napierala M. (2022). Selected histone deacetylase inhibitors reverse the frataxin transcriptional defect in a novel friedreich’s ataxia induced pluripotent stem cell-derived neuronal reporter system. Front. Neurosci. 16, 836476. 10.3389/fnins.2022.836476 35281493 PMC8904878

[B71] Schreiber A. M. Misiorek J. O. Napierala J. S. Napierala M. (2019). Progress in understanding friedreich's ataxia using human induced pluripotent stem cells. Expert Opin. Orphan Drugs 7 (2), 81–90. 10.1080/21678707.2019.1562334 30828501 PMC6392065

[B72] Shan B. Xu C. Zhang Y. Xu T. Gottesfeld J. M. Yates J. R. 3rd. (2014). Quantitative proteomic analysis identifies targets and pathways of a 2-aminobenzamide HDAC inhibitor in Friedreich's ataxia patient iPSC-derived neural stem cells. J. Proteome Res. 13 (11), 4558–4566. 10.1021/pr500514r 24933366 PMC4227551

[B73] Sharma R. Bhatti S. Gomez M. Clark R. M. Murray C. Ashizawa T. (2002). The GAA triplet-repeat sequence in Friedreich ataxia shows a high level of somatic instability *in vivo*, with a significant predilection for large contractions. Hum. Mol. Genet. 11, 2175–2187. 10.1093/hmg/11.18.2175 12189170

[B92] Shen X. Beasley S. Putman J. N. Li Y. Prakash T. P. Rigo F. (2019). Efficient electroporation of neuronal cells using synthetic oligonucleotides: identifying duplex RNA and antisense oligonucleotide activators of human frataxin expression. RNA 25 (9), 1118–1129. 10.1261/rna.071290.119 31151992 PMC6800520

[B74] Simon D. Seznec H. Gansmuller A. Carelle N. Weber P. Metzger D. (2004). Friedreich ataxia mouse models with progressive cerebellar and sensory ataxia reveal autophagic neurodegeneration in dorsal root ganglia. J. Neurosci. 24 (8), 1987–1995. 10.1523/JNEUROSCI.4549-03.2004 14985441 PMC6730414

[B75] Soldner F. Jaenisch R. (2018). Stem cells, genome editing, and the path to translational medicine. Cell 175, 615–632. 10.1016/j.cell.2018.09.010 30340033 PMC6461399

[B76] Strawser C. J. Schadt K. A. Lynch D. R. (2014). Therapeutic approaches for the treatment of Friedreich’s ataxia. Expert Rev. Neurother. 14, 949–957. 10.1586/14737175.2014.939173 25034024

[B77] Takahashi K. Yamanaka S. (2006). Induction of pluripotent stem cells from mouse embryonic and adult fibroblast cultures by defined factors. Cell 126, 663–676. 10.1016/j.cell.2006.07.024 16904174

[B78] Terzi E. M. Sviderskiy V. O. Alvarez S. W. Whiten G. C. Possemato R. (2021). Iron-sulfur cluster deficiency can be sensed by IRP2 and regulates iron homeostasis and sensitivity to ferroptosis independent of IRP1 and FBXL5. Sci. Adv. 7 (22), eabg4302. 10.1126/sciadv.abg4302 34039609 PMC8153722

[B79] Turchi R. Faraonio R. Lettieri-Barbato D. Aquilano K. (2020). An overview of the ferroptosis hallmarks in friedreich's ataxia. Biomolecules 10 (11), E1489. 10.3390/biom10111489 33126466 PMC7693407

[B80] Vannocci T. Manzano R. N. Beccalli O. Bettegazzi B. Grohovaz F. Cinque G. (2018). Adding a temporal dimension to the study of Friedreich’s ataxia: the effect of frataxin overexpression in a human cell model. Dis. Model. Mech. 11, dmm032706. 10.1242/dmm.032706 29794127 PMC6031361

[B81] Vázquez-Manrique R. P. González‐Cabo P. Ros S. Aziz H. Baylis H. A. Palau F. (2006). Reduction of *Caenorhabditis elegans* frataxin increases sensitivity to oxidative stress, reduces lifespan, and causes lethality in a mitochondrial complex II mutant. FASEB J. 20, 172–174. 10.1096/fj.05-4212fje 16293572

[B82] Vicente-Acosta A. Gimenez-Cassina A. Diaz-Nido J. Loria F. (2022). The smoothened agonist SAG reduces mitochondrial dysfunction and neurotoxicity of frataxin-deficient astrocytes. J. Neuroinflammation 19 (1), 93. 10.1186/s12974-022-02442-w 35413853 PMC9006607

[B83] Virmouni S. A. Sandi C. Al-Mahdawi S. Pook M. A. (2014). Cellular, molecular and functional characterisation of YAC transgenic mouse models of friedreich ataxia. PLoS ONE 9, e107416. 10.1371/journal.pone.0107416 25198290 PMC4157886

[B84] Wong A. Yang J. Cavadini P. Gellera C. Lonnerdal B. Taroni B. (1999). The Friedreich’s ataxia mutation confers cellular sensitivity to oxidant stress which is rescued by chelators of iron and calcium and inhibitors of apoptosis. Hum. Mol. Genet. 8 (3), 425–430. 10.1093/hmg/8.3.425 9949201

[B85] Wong A. O. Wong G. Shen M. Chow M. Z. Tse W. W. Gurung B. (2019)., Correlation between frataxin expression and contractility revealed by *in vitro* friedreich's ataxia cardiac tissue models engineered from human pluripotent stem cells Stem Cell Res. Ther.1. 10. PMCID, 203PMC6615274. PMID: 31286988. 10.1186/s13287-019-1305-y 31286988 PMC6615274

[B86] Xu L. Sun Z. Xing Z. Liu Y. Zhao H. Tang Z. (2022). Cur@SF NPs alleviate friedreich's ataxia in a mouse model through synergistic iron chelation and antioxidation. J. Nanobiotechnology 20 (1), 118. 10.1186/s12951-022-01333-9 35264205 PMC8905737

[B93] Zhang S. Napierala M. Napierala J. S. (2019). Therapeutic Prospects for Friedreich's Ataxia. Trends Pharmacol. Sci. 40 (4), 229–233. 10.1016/j.tips.2019.02.001 30905359 PMC6826337

